# A possible theranostic approach of chitosan-coated iron oxide nanoparticles against human colorectal carcinoma (HCT-116) cell line

**DOI:** 10.1016/j.sjbs.2021.08.078

**Published:** 2021-08-28

**Authors:** Abdullah A. Alkahtane, Hamzah A. Alghamdi, Alanoud T. Aljasham, Saad Alkahtani

**Affiliations:** Department of Zoology, College of Science, King Saud University, Riyadh, Saudi Arabia

**Keywords:** Colorectal cancer, Photodynamic therapy, Reactive oxygen species, Metallic nanoparticles, MRI

## Abstract

Iron oxides have become increasingly popular for their use as a diagnostic and therapeutic tool in oncology. This study aimed to improve pharmacological valuable of Fe_3_O_4_, which may be use to diagnosis colorectal cancers (CRC). Here, we have developed chitosan (CS) coated Fe_3_O_4_ through a cost-effective procedure. First, we determined the characterization of OA-C-Fe_3_O_4_ by FTIR, UV–Vis spectra, and TEM. Then, we evaluated the photodynamic therapeutic (PDT) activity of OA-C-Fe_3_O_4_ in human colorectal carcinoma cell lines (HCT 116). Current results revealed that the light-induced enhanced reactive oxygen species (ROS) activity of the nanoparticles (NPs) and caused cell death *via* the activity of caspase 9/3. The *in vitro* magnetic resonance imaging (MRI) experiments in (HCT 116) and human embryonic kidney cells (HEK 293) illustrated that nanohybrid is an effective MRI contrasting agents for the diagnosis of colorectal cancer.

## 1 Introduction

The early-stage treatment regimens with appropriate light-sensitive therapeutic modalities play a crucial role in reducing the metastatic tumors of colorectal cancer (CRC) (Chakrabarti et al., 2020, Rawla et al., 2019). One of the versatile sources for treating CRC is photodynamic therapy (PDT) because of its non-invasive and permits treatment of large areas (Nafiujjaman et al., 2015, Wang et al., 2014). This therapy depends on the irradiation of UV light with photosensitizer (PS) that, upon photoexcitation, gives rise to ROS in target tissues, leading to cell death (Castano et al., 2006, Yan et al., 2019). In recent years, there has been considerable attention in developing new metallic nanomedicines as a PDT agent for the treatment of metastatic CRC at cellular, subcellular and molecular level (Yan et al., 2021, Rampado et al., 2019). At the time of PDT, the systemic administration of the photosensitizing light is delivered to the site of tumor growth, which has deeper tissue penetration than visible wavelengths. It would therefore shown to be the safest route for treating metastatic CRC lesions (Cabeza et al., 2020; (Iranpour et al., 2021). The specific delivery of a drug during the course of PDT is the most important phenomena for the therapeutic efficacy because of biomolecular oxidation, triggering the alteration in gene expression regulation (Castano et al., 2006, Pan et al., 2007, Penon et al., 2016, Weinstein et al., 2010). Compared to conventional therapy, PDT causes less toxicity to healthy cells, since ROS are produced only in the presence of monochromatic light (Sai et al., 2021, Zhou et al., 2016). PDT-based drugs offer potential benefits over chemotherapy and radiotherapy, which act as curative potential towards a wide range of malignant diseases (Hu et al., 2020). However, few limitations such as tumour selectivity, shorter ROS lifetime, and minute laser penetrability disrupt their clinical applications (Allison et al., 2008, Li et al., 2014). To overcome these limits, it is essential to search for new schemes based on nanoparticulate platforms in order to boost the defined localization of PS (Yan et al., 2018). It has been documented that the therapeutic prospective of PDT could be altered by formulating PS into a nanoparticulate system (Niculescu and Grumezescu, 2021, Obaid et al., 2016). The promising pharmacokinetics, biodistribution, and solubility displayed by NPs once combined with photosensitizers could afford a significant understanding of the drug's localization (Liu et al., 2017, Patra et al., 2018).

Metallic NPs have been increasingly highlighted due to their subsequent use as diagnostic and drug vehicle tools in anti-cancer and antibacterial therapy (Kuchur et al., 2020). Researchers have investigated various NPs including iron oxide NPs (Fe_3_O_4)_, which have exhibited favourable characteristics such as biocompatibility, low toxicity, and aqueous solubility (Dadfar et al., 2019, Xu et al., 2019). Furthermore, these NPs generate ROS responsible for DNA damage and eventually cell death, making them an essential tool in cancer theranostics (Dadfar et al., 2019). On the contrary, bulky aggregates of pure Fe_3_O_4_ NPs exhibit strong dipole–dipole interactions between particles, which may hamper their biocompatibility (Nandi et al., 2017). Chitosan (CS) polysaccharides have been frequently undertaken to modify metal NPs' behaviour, due to their excellent biocompatibility, degradability, and low toxicity. Therefore, it has been acknowledged as a favourable tool for tumour visualization using confined magnetic field gradients (Xie et al., 2018). However, CS-coated magnetic NPs are likely to be adsorbed by normal tissues through their circulation route due to their mucoadhesive and bioadhesive features (Arias et al., 2020, Vieira et al., 2019). Researchers have recently shown that CS-iron oxide magnetic NPs coated with phytic acid (PTA) displayed greater cytotoxicity than PTA alone on HT-29 colon cancer cells, leaving healthy cells unaffected (Barahuie et al., 2017).

Herein, we developed a CS coated Fe_3_O_4_ (represented as OA-C-Fe_3_O_4_), characterized it, and evaluated its PDT efficacy along with its diagnostic application in colorectal cancer.

## 2 Experimental section

### 2.1 Chemicals and cell lines

Iron salts (FeCl_2_·4H_2_O and FeCl_3_·6H_2_O), sodium dihydrogen phosphate dehydrate (NaH_2_PO_4_·2H_2_O), oleic acid (OA), chitosan (CS), dichlorofluorescein diacetate (DCFH-DA), and NaOH were purchased from Sigma Aldrich, USA. Both HCT 116 and HEK 293 cell lines were obtained from ATCC, USA. Culture Medium (DMEM), antibiotic, fetal bovine serum (FBS) and EDTA were procured from Gibco-Life Technologies (Grand Island, NY, USA). Antibodies were purchased from Santa Cruz Biotechnology, Inc. USA and eBioscience, Inc. San Diego, USA. MTT and DAPI were purchased from Thermo Fisher Scientific (USA).

### 2.2 Synthesis of OA-Fe_3_O_4_ nanoparticles

The Fe_3_O_4_ NPs were synthesized according to a previously described procedure (Gupta et al., 2019). In this method, aqueous solution (100 mL) of FeCl_2_·4H_2_O (0.8 g) and FeCl_3_·6H_2_O (2.0 g) were refluxed followed by stirring at 80 °C. To this mixture, 20 mL of ammonia solution (NH_4_OH, 25 wt%) was added slowly under dynamic stirring for 20 min to increase the number and size of the nanoparticles. The surface alteration of NPs takes place by adding oleic acid (OA) (7 mL) into the above suspension at 75 °C for 40 min. The suspended solution was cooled, followed by cleaning with ice-cold water and ethanol to eliminate any impurities and reactants. The prepared OA-Fe_3_O_4_ NPs were stored in a vacuum desiccator overnight earlier to their use. Applying the co-precipitation of anhydrous iron salts in the presence of chitosan, tailor-made chitosan-coated Fe_3_O_4_ NPs were obtained (Mohammadi et al., 2014). Chitosan (0.15 g) was solidified using 1% acetic acid (30 mL) at 4.8 pH (maintaining by addition of NaOH). The OA-Fe_3_O_4_ NPs (2 g) were stirred with chitosan solution (50 mL) under an inert atmosphere at 50 °C. Ammonia solution (40 mL) was gently supplemented to the reaction mixture to yield the desired NPs after stirring for 30 min. The colloidal OA-C- Fe_3_O_4_ was continuously washed with water and separated by a magnetic decantation process.

### 2.3 Characterizations

For TEM analysis, 5 mg of OA-C-Fe_3_O_4_ was dispersed into phosphate buffer saline (PBS) for 5 min under sonication. Air-dried sample of the dispersed solution was subjected to the carbon-coated copper grid to perform the measurement (Roy et al., 2014). The samples' FT-IR spectra were recorded using KBr pellets in a Perkin Elmer IR 783 spectrophotometer (Lesiak et al., 2019) and UV–Visible spectrum recorded on Shimadzu UV-1650.

### 2.4 Cell culture and MTT assay

HCT 116 and HEK 293 cells were maintained in DMEM medium containing 10% FBS and 1% PSN under humidified atmosphere (5% CO_2_) at 37 °C. Once 75–80% confluence was achieved, cells were re-equilibrated with trypsin (0.25%) and EDTA (0.52 mM) in PBS and harvested at the appropriate density for 24 h before cell viability (Larsson et al., 2020, Segeritz and Vallier, 2017). In this assay, the HCT 116 cells were treated with various concentrations of OA-C- Fe_3_O_4_ (0–60 µg/mL) for 24 h. A similar assay was also accompanied in HEK-293 cell line for measuring the toxicity in a normal cell line. Cells were rinsed with PBS, and MTT solution was added to form formazan salt. Then, the media was replaced with DMSO (200 µL) to dissolve the formed formazan crystal in each well. The proliferation of cells was determined spectrophotometrically at 595 using an ELISA reader (Emax, Molecular device, USA) and the cell viability measurement as follows:

$$Cell\;viability\;\left(\%\right)=\frac{\mathrm{OD}\;of\;Control-OD\;of\;treated}{\mathrm{OD}\;of\;Control}\times 100$$

### 2.5 In vitro photodynamic therapy

According to a preliminary screening, the experiment was conducted on HCT 116 cells. A particular concentration of OA-C-Fe_3_O_4_ (20 µg/mL) was opted to determine its photosensitizing impact within the cells. Furthermore, OA-C-Fe_3_O_4_ treated HCT 116 cells were exposed to blue light over a period of time (0–40 min) to measure the ability of photodynamic therapy (Wang et al., 2020).

### 2.6 Apoptosis using flow cytometry

Annexin V-FITC detection kit was used to recognize apoptosis by staining phosphatidylserine molecules (Lassus and Hibner, 1998). At first, cells were treated with 20 µg/mL of OA-C-Fe_3_O_4_ and further placed under blue light at different time (0, 10, and 20 min). A flow cytometer (BD LSRFortessa TM San Jose, CA, USA) was used to analyse the expression of Annexin V-FITC by employing Flow Jo (Version 10.0) software.

### 2.7 Reactive oxygen species (ROS) measurement

The intracellular reactive oxygen species (iROS) was measured by quantifying the green intensity of the cell-permeable fluorogenic probe 2́,7́-dichlorofluorescein diacetate (H2DCFDA) with reference to the untreated control (Redza-Dutordoir and Averill-Bates, 2016). A medium comprising H2DCFDA where the treated cells (4 × 10^3^) were resuspended and for 30 min at 37 °C. The results obtained were examined using an argon laser at 488 nm by BD LSRFortessa flow cytometer (Becton Dickinson, Franklin Lakes, NJ, USA).

### 2.8 Immunofluorescence

HCT 116 cells (control and treated) were washed twice in PBS and incubated with a blocking buffer containing bovine serum (2%) and Triton X-100 (0.3%) for 1 h. Further, the cells were fixed with primary antibodies specific (p53, p21 and gH2AX) at 4 °C overnight. After washing with PBS, the cells were incubated with secondary antibodies (anti-mouse/anti-rabbit FITC and PE). The stained cells were counterstained with DAPI and mounted with the ProLong antifade reagent (Molecular Probe, Eugene, OR, USA), then they were imaged using confocal microscopy (FV 10i, Olympus, Japan).

### 2.9 Caspase-3 and -9 activities

Using commercially available assay kits for caspase-3 and caspase-9, an ELISA reader was undertaken to achieve this spectrophotometric assay at 405 nm (Das et al., 2009) as per the manufacturer's instructions (BioVision Research Products, Mountain View, CA).

### 2.10 MRI study

In this imaging analysis, optimum numbers of HEK 293 and HCT 116 cells (6 × 10^3^) were treated with nanoparticles at two different concentrations (5 and 10 µg/ mL) and incubated for 8 h. After that, cells were thoroughly cleaned with PBS and fixed with 4% paraformaldehyde. To circumvent air susceptibility, low melting agarose (100 mL of 2%) was added to each well and the plate was preserved at 4 °C to solidify cell suspensions. The samples placement was implemented on a 3 T clinical MRI scanner (Siemens MAGNETOM Verio) and MR phantom images were visualized by using a spin-echo multi-section pulse sequence. Several defaults parameters (echo times: 13.2–212.8 ms, repetition time: 1770 ms, acquisition matrix: 208 mm × 230 mm and section thickness: 3 mm) were used to collect the coronal images in order to determine the transverse relaxation (T2) of the sample.

### 2.11 Statistical analysis

All the measured data were executed using OriginPro 8.0 software (San Diego, CA, USA). Statistical significance and difference among set conditions were evaluated via one-way analysis of variance (ANOVA). The observed data with a *p*-value < 0.05 were considered as statistically significant.

## 3 Result

### 3.1 Characterizations

**Fig. 1:**
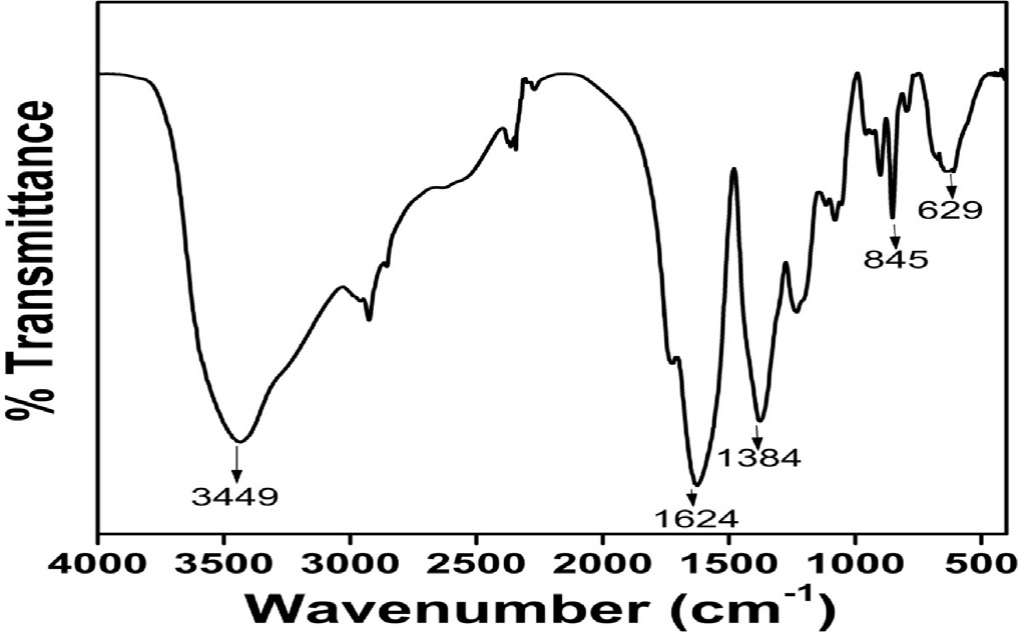
FTIR data of OA-C-Fe_3_O_4_ nanoparticles.

**Fig. 2:**
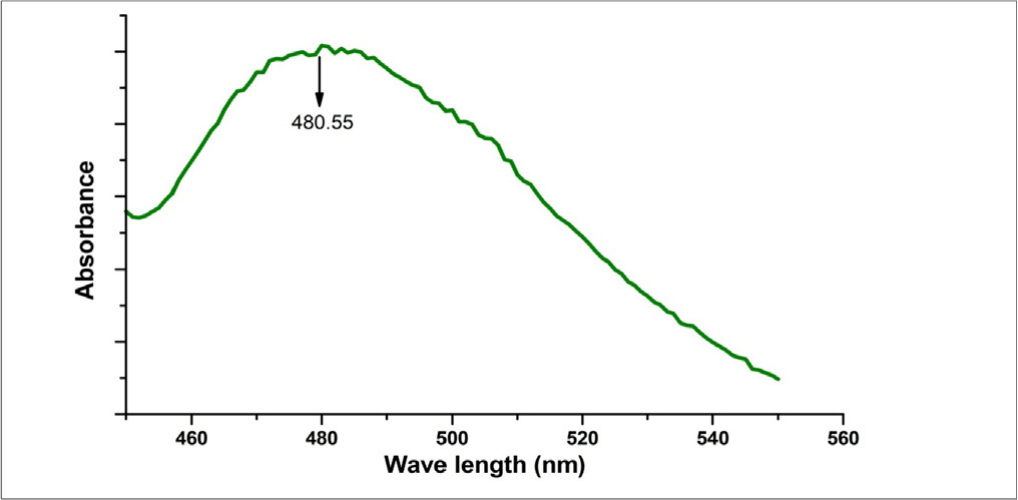
UV–Vis data of OA-C-Fe_3_O_4_ nanoparticles.

**Fig. 3:**
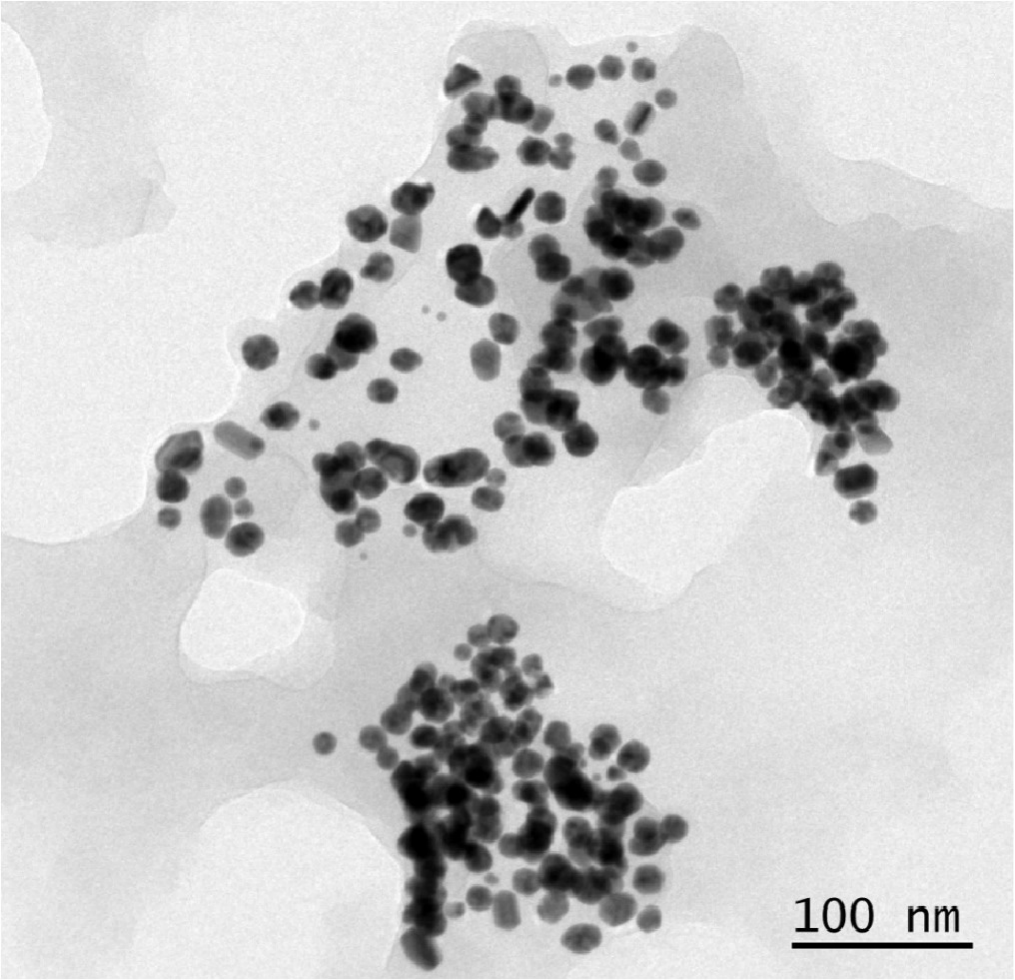
TEM image of OA-C- Fe_3_O_4_ nanoparticles under 100 nm scale bar.

The FTIR data confirmed that the characteristics O-H stretching of the hydroxyl group present in the oleic acid was observed at 3449 cm^−1^. The peaks observed at 1384, 1624 cm^−1^ corresponded to C-O, C = O respectively. Another small peak was displayed around 1624 cm^−1^ for C-H stretching vibrations, while a peak observed at 629 cm^−1^ was ascribed to the Fe-O stretching vibration. However, these reported data result from measurements in a polar environment of the KBr pellet, which may shift the peak position. This FTIR data (Fig. 1) confirmed the structure of OA-C-Fe_3_O_4._ The electronic spectrum of OA-C-Fe_3_O_4_ was perceived under UV–Vis spectrophotometer (Fig. 2), which displayed the primary peak position at 480 nm, and hence this wavelength has been preferred for further biological studies. The morphological characterization of OA-C-Fe_3_O_4_ has been obtained using TEM (Fig. 3), which showed a branched network-like appearance having a segment length of 300–500 nm and width of 350–450 nm. The morphology of OA-C-Fe_3_O_4_ exhibits a nano-egg-like property with a maximum length and width of 15 and 5 nm, respectively without any agglomeration was observed under the microscope.

### 3.2 Estimation of cytotoxicity

**Fig. 4:**
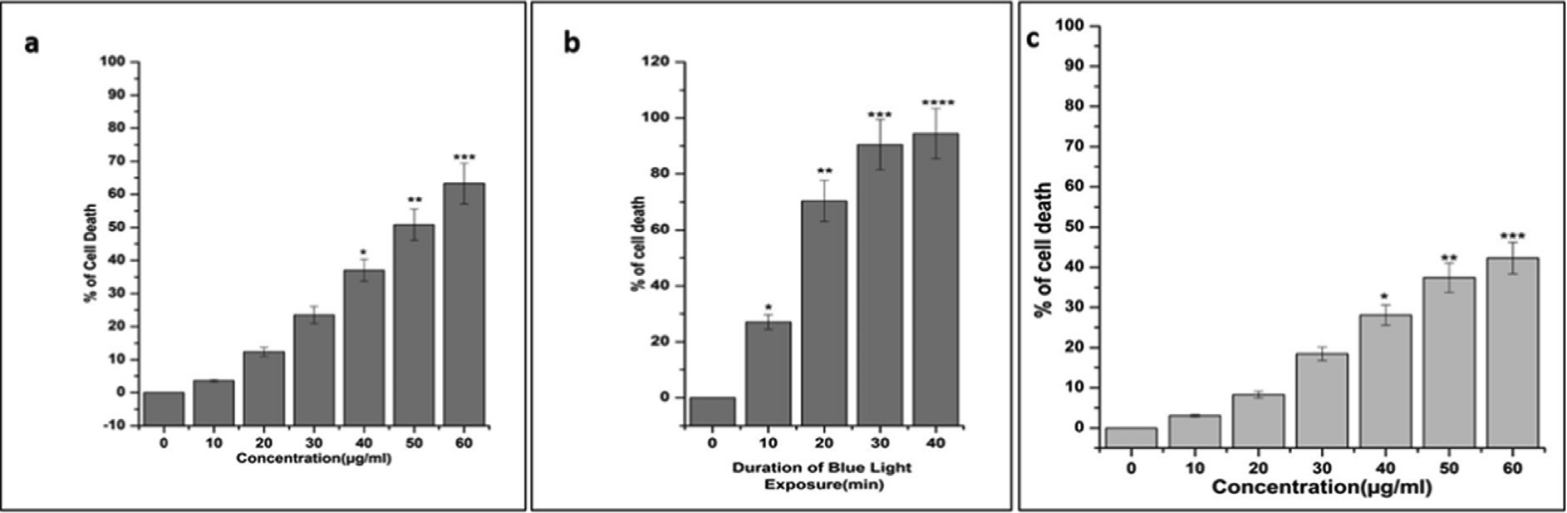
(a). MTT assay data of OA-C- Fe_3_O_4_ nanoparticles (0–60 µg/mL) with HCT 116. Each value signifies the mean ± SE of three trials, n = 3, (*p < 0.05, **p < 0.01, ***p < 0.001) compared with the control; (b) MTT assay data after 10–40 min blue light exposure (after 20 µg/mL OA-C- Fe_3_O_4_ treatment); (c) MTT assay data of OA-C- Fe_3_O_4_ nanoparticles (0–60 µg/mL) with HEK-293.

To determine the cytotoxicity of nanohybrid (OA-C-Fe_3_O_4_) on HCT 116 cells, MTT assay was used without being exposed to blue light (Fig. 4a). Upon treatment of various amount of OA-C-Fe_3_O_4_ (0–60 µg/mL) with HCT 116 cells (Fig. 4a), there was no significant cytotoxicity observed up to 30 µg/mL. In contrast, most of all the cells died at 55 µg/mL of OA-C-Fe_3_O_4_. Under blue light exposure, the photodynamic effects of OA-C-Fe_3_O_4_ were detected which is comparable with HCT 116 cells being treated with 20 µg/mL of OA-C-Fe_3_O_4_ before blue light irradiation at various exposure time (10–40 mins). As shown in (Fig. 4b), the data shows significant cell death at a non-toxic concentration of OA-C-Fe_3_O_4_ in the presence of blue light irradiation. The cell viability has been decreased correspondingly with respect to the blue light irradiation duration in presence of 20 µg/ml of OA-C-Fe_3_O_4_. For further cell biological experiments, 10 and 20 min of blue light exposure duration was designated. The cytotoxicity of OA-C-Fe_3_O_4_ was also tested in normal HEK-293 cell line (Fig. 4c). The cell viability data cleared that, there was no such cytotoxic effect up to 30 µg/mL concentration of OA-C-Fe_3_O_4_.

### 3.3 Measurement of apoptosis

**Fig. 5:**
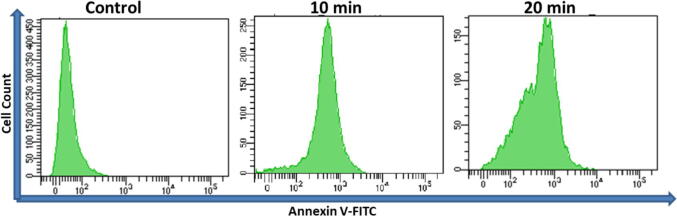
Annexin V-FITC data after 10 and 20 min of blue light exposure (20 µg/ml OA-C- Fe_3_O_4_ nanoparticles treated HCT 116 cell line).

Phosphatidylserine (PS) is a phospholipid unit of cell membrane covering and externalized throughout apoptosis. Annexin-V is a PS-binding protein used to detect apoptotic cells when attached to a specific fluorophore (FITC). The fluorescence intensity of FITC was found to be increased on exposure to blue light at different time intervals 10 and 20 min (Fig. 5). The results indicate that the source of apoptotic cell death was prompted by light irradiation on 20 µg/mL of OA-C-Fe_3_O_4_.

### 3.4 Measurement of ROS

**Fig. 6:**
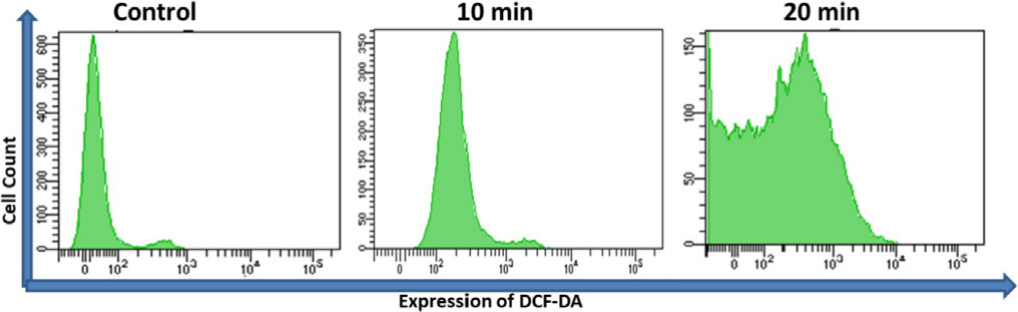
Expression of ROS generation after 10 and 20 min of blue light exposure (20 µg/ml OA-C- Fe_3_O_4_ nanoparticles treated HCT 116 cell line).

ROS generation play a vital role in the initiation of apoptosis and hence, is counted as to determine apoptosis in the cell. Upon 20 µg/mL of OA-C-Fe_3_O_4_ treatment, the mean emission profile of DCF dye was significantly (p < 0.05) increased once irradiated on a blue light in appropriate amount of time (Fig. 6). After multiple times examination, we found that the relative DCF intensity plots increased over time.

### 3.5 Immunofluorescence

**Fig. 7:**
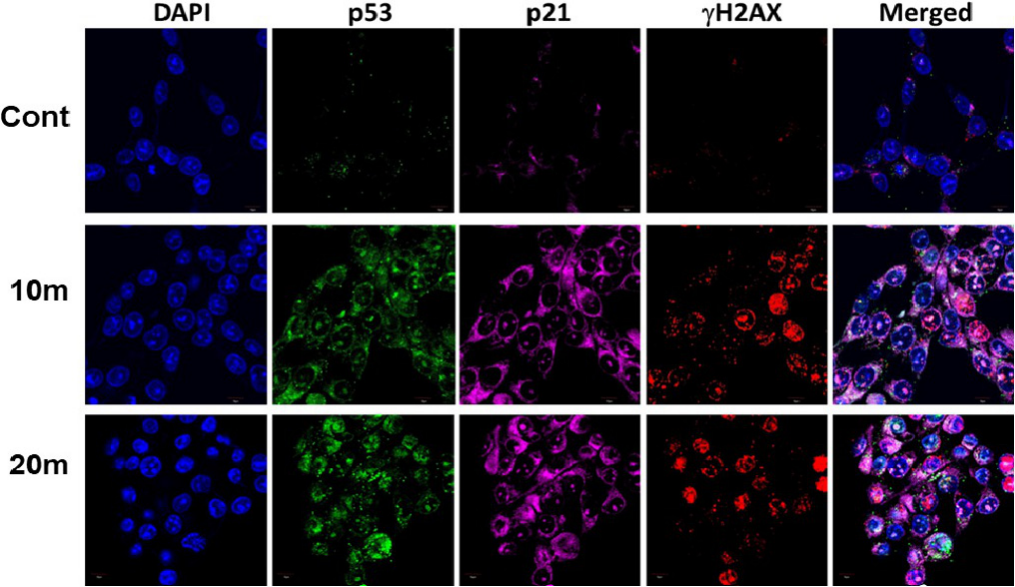
Expression of p53, p21 and γH2AX after 10 and 20 min of light exposure (20 µg/mL OA-C- Fe_3_O_4_ nanoparticles treated HCT 116 cell line).

Several studies indicate a direct correlation between DNA damage and apoptosis. DNA damage is accountable for initiating apoptosis by stimulating numerous signalling pathways (Roos & Kaina, 2013). H2AX is a form of the H2A protein family and is a fragment of the histone octamer found at the centre of a nucleosome core particle (Kuo and Yang, 2008). Upon DNA damage by exogenous and endogenous sources, H2AX is phosphorylated at Ser139 to form γH2AX (Rogakou et al., 1998), which in turn activate the tumor suppressor p53, leading to transient expression of the cyclin-dependent kinase inhibitor (CKI) p21. This either triggers momentary G1 cell cycle arrest or leads to a chronic state of senescence or apoptosis, a form of genome guardianship (Fragkos et al., 2009). In HCT 116 cells treated with OA-C-Fe_3_O_4_ (20 µg/mL), the expression of p53 and p21 was determined after 10 and 20 min of due course of light. We observed that after the OA-C-Fe_3_O_4_ treatment, both p53 and p21 expression have been increased compared to the control (Fig. 7).

### 3.6 Expression of caspase-3 and −9 activity

**Fig. 8:**
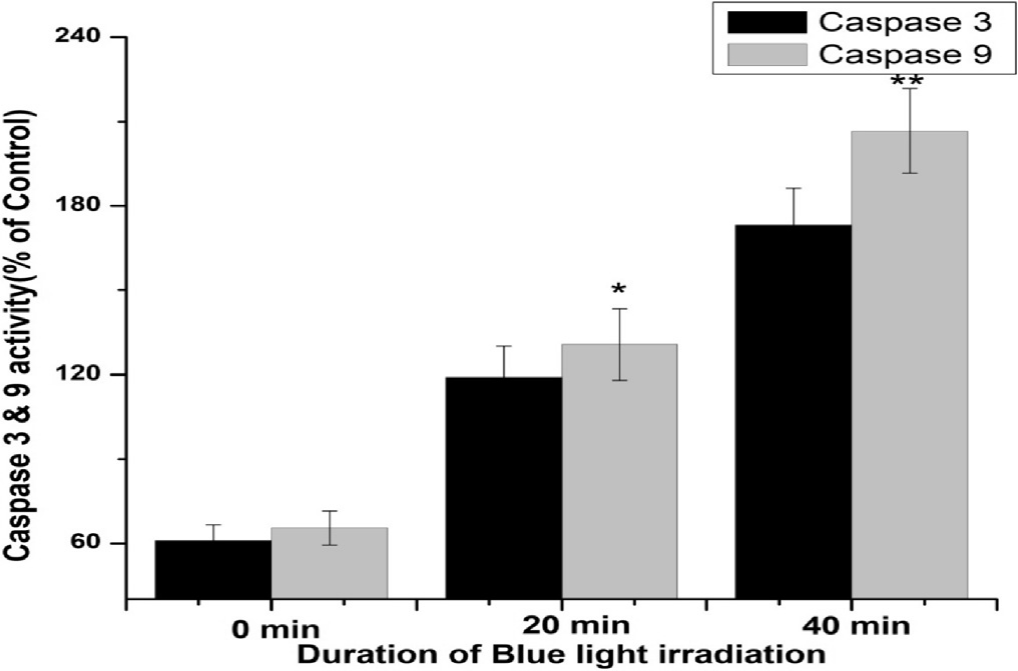
Expression of Caspase 3 and 9 after 10, 20 and 40 min of light exposure (20 µg/mL OA-C-Fe_3_O_4_ nanoparticles treated HCT 116 cell line). Each value represents the mean ± SE of three experiments, n = 3, (*p < 0.05, **p < 0.01) compared with the control.

Cytochrome *c* is a crucial component of the intrinsic apoptotic pathway. Apoptotic stimuli trigger mitochondria to release *cytochrome c*, resulting in caspase activation and apoptosis. In this study, cytochrome *c* was simultaneously enhanced by blue light irradiation on HCT 116 cells treated with 20 µg/mL of OA-C-Fe_3_O_4_. Caspases-9 and caspase-3 were the final determinants of apoptosis (Fig. 8), which specified that OA-C-Fe_3_O_4_ treatment leads to apoptosis in a time-dependent manner under blue light.

### 3.7 MRI study

**Fig. 9:**
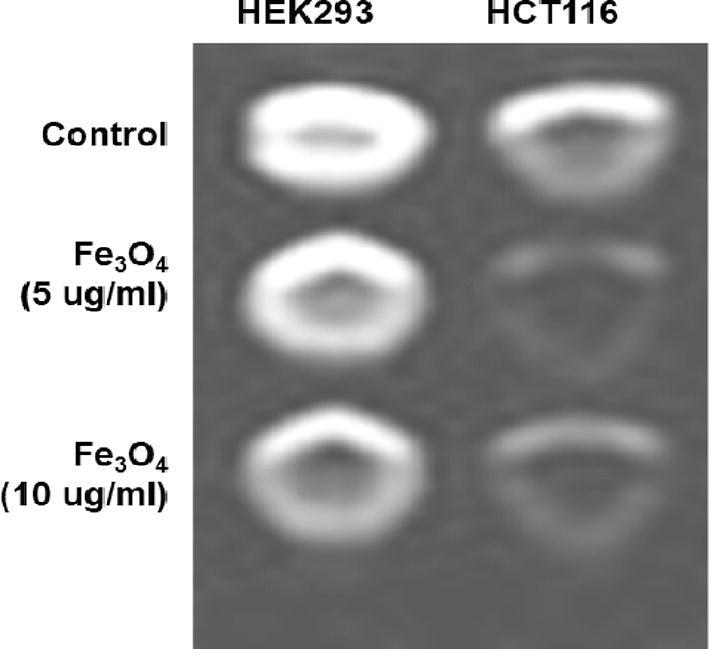
T2 weighted MRI phantom images of HEK 293 and HCT 116 cells treated with different concentrations of OA-C-Fe_3_O_4_ treatment.

MRI studies have been carried out to develop a theranostics application of the synthesized NPs. In addition to its therapeutic properties, OA-C-Fe_3_O_4_ has potential MRI contrasting ability in HCT 116 cells. In Fig. 9, The T2 weighted MRI phantom images show that HCT 116 has better contrast than HEK 293. Thus, OA-C- Fe_3_O_4_ offers enhanced ROS activity and MRI contrast ability, both of which may be used for diagnostics and treatment of colorectal cancer.

## 4 Discussions

Colorectal cancer is one of the most prevalent cancer types that affect millions of people every year. Early diagnosis of cancer increases the chances of survival and recovery. Iron oxide NPs have attracted considerable interest due to their superparamagnetic properties and their potential biomedical applications arising from their biocompatibility and non-toxicity (Khayat Sarkar and Khayat Sarkar, 2013). FT-IR spectra allowed scientists to understand the local molecular environment of organic molecules on the surface of NPs. In the present work, FTIR spectra were recorded using KBr pellets in the frequency range of 4000–400 cm^−1^. The FTIR spectra indicated absorption bands due to O-H stretching, C-O bending, C-H stretching, and Fe-O stretching vibrations. There is consistency between the observed spectra and those previously published by different investigators. The coating of chitosan is established by the appearance of the peak at 1624 cm^−1^ considered to be C-H stretching vibrations. Besides, the stretch vibration of C–O was found at 1384, 1624 cm^−1^. The Fe-O stretching backbone vibrations peak was shifted to 629 cm^−1^ for the OA-C-Fe_3_O_4_ compared to the uncoated Fe_3_O_4_, which is consistent with the previous studies (Li et al., 2015: Zeinali et al., 2016). Peaks at 3449 cm^−1^due to the O-H stretching model adsorbed on the surface of the Fe_3_O_4_ NPs (Zhang et al., 2013). Altogether, the peaks from OA-C-Fe_3_O_4_ NPs indicated that the chitosan effectively coated the Fe_3_O_4_ NPs. This binding was also confirmed by UV–Vis Spectrophotometry which showed a peak at 480 nm (Fig. 2), considering another evidence for binding of chitosan on the Fe_3_O_4_ NPs surfaces. The TEM images of OA-C-Fe_3_O_4_ NPs reveal that the NPs are spherical, with a maximum length and width of 15 and 5 nm, respectively. It was cleared that the chitosan prevents the aggregation of NPs so, OA-C-Fe_3_O_4_ NPs had essentially low dispersion, compared to the naked NPs (Zeinali et al., 2016). The cytotoxicity of OA-C-Fe_3_O_4_ NPs was evaluated against HCT 116 cell and HEK-293 cell lines. It was observed that cell death is directly proportional to the concentrations of OA-C-Fe_3_O_4_ NPs. However, in the presence of blue light irradiation at different exposure times (10–40 mins), the data shows significant cell death at a non-toxic concentration of OA-C- Fe_3_O_4_, with 10 and 20 min being the most optimum time for future experiments. These data suggest that the chitosan coating reduces the toxic effects of Fe_3_O_4_ NPs, which may be attributed to control the release of Fe2^+^ ions, triggering ROS-mediated cell death (Shukla et al., 2015). Early apoptotic cells can be identified by green fluorescence of annexin V-FITC, as it has a high affinity towards PS residues externalized from the inner to the outer leaflet of the plasma membrane during early stages of apoptosis (Koopman et al., 1994). Results of flow cytometric analysis of annexin V-FITC stained HCT 116 cells treated with 20 µg/ml OA-C- Fe_3_O_4_ after 10 and 20 min of blue light exposure. The results establish the efficient induction of apoptotic cell death in HCT 116 cells by OA-C- Fe_3_O_4_ in a dose-dependent manner under light irradiation. ROS production plays a vital role in the initiation of apoptosis and hence, is counted as to determine apoptosis in the cell (Redza-Dutordoir and Averill-Bates, 2016). DCF-DA assay for ROS generation analysis revealed that DCF production is high in OA-C- Fe_3_O_4_ treated HCT 116 cells concerning the untreated control. Production of highly fluorescent DCF in OA-C- Fe_3_O_4_ treated HCT 116 cells increased under blue light irradiation over time which is consistent with previous studies (Shukla et al., 2015). Analysis of immunofluorescence staining indicates that the expression of γH2AX, p53 and, p21 in the presence of blue light is upregulated following the treatment of OA-C- Fe_3_O_4_. Therefore, it can be concluded that the light-induced DNA damage governed by OA-C- Fe_3_O_4_ is mainly due to the production of ROS as a result of p53/21 activation. The Final determinant of apoptosis was via the activation of Caspases-9 and caspase-3, induced by the release of Cytochrome *c* from mitochondria (Arnoult et al., 2002). Caspase-9 and 3, which play a crucial role in the apoptotic pathway of cells, were induced following treatment with CS- Fe_3_O_4_ NPs. When HCT 116 cells were treated with 20 µg/mL concentration of OA-C- Fe_3_O_4_ NPs for 0, 20, and 40 mins, the activity of caspase-9/3 was increased in a concentration and time dependent manner. A similar result was obtained in a previous study carried out on human breast cancer cells (MCF-7). MRI data showed that OA-C- Fe_3_O_4_ offers enhanced ROS activity and MRI contrast ability, both of which may be used for diagnostics and treatment of colorectal cancer.

## 5 Conclusion

This study has established easy and cost-effective synthesis methodologies of a novel nanohybrid (OA–C-Fe_3_O_4_) that has shown anti-cancer activity in the human colorectal cancer cell line and high contrast abilities in MRI images.

## CRediT authorship contribution statement

Abdullah Alkahtane and Hamzah Alghamdi were performed the cell culture, treatments and preparation of nanoparticles. Hamzah Alghamdi was performed the photodynamic therapy. Saad Alkahtani and Abdullah Alkahtane were performed apoptotic and oxidative stress parameters. Hamzah Alghamdi and Saad Alkahtani were performed MRI study and statistical analysis. Alanoud Aljasham and SaadAlkahtani were involved in the conception and design of the study, data interpretation, and critically revised the manuscript. All authors read and approved the final manuscript.

## Declaration of Competing Interest

The authors declare that they have no known competing financial interests or personal relationships that could have appeared to influence the work reported in this paper.
